# Circulating extracellular vesicles during pregnancy in women with type 1 diabetes: a secondary analysis of the CONCEPTT trial

**DOI:** 10.1186/s40364-021-00322-8

**Published:** 2021-09-06

**Authors:** Akram Abolbaghaei, Marc-André Langlois, Helen R Murphy, Denice S. Feig, Dylan Burger, on behalf of the CONCEPTT Collaborative Group

**Affiliations:** 1 Chronic Disease Program, Kidney Research Centre, Ottawa Hospital Research Institute, Ottawa, Canada; 2 Departments of Medicine and Cellular and Molecular Medicine, University of Ottawa, 2513-/451 Smyth Road, Ontario K1H 8M5 Ottawa, Canada; 3 Department of Biochemistry, Microbiology and Immunology, Faculty of Medicine, University of Ottawa, Ottawa, Canada; 4 Department of Women and Children’s Health, St Thomas’ Hospital, King’s College London, London, UK; 5 Wolfson Diabetes and Endocrine Centre, Cambridge University Hospitals NHS Foundation Trust, Cambridge, UK; 6 Department of Medicine, University of East Anglia, Norwich, UK; 7 Department of Medicine, University of Toronto, Toronto, ON Canada; 8 Sinai Health System, Toronto, ON Canada; 9 Lunenfeld-Tanenbaum Research Institute, Toronto, ON Canada

**Keywords:** Extracellular vesicle, Type 1 diabetes, Pregnancy, Endothelial, Platelet, Microparticle, Microvesicle, Biomarker

## Abstract

**Background:**

Extracellular vesicles are membrane vesicles that are released into the extracellular environment and accumulate in the circulation in vascular disease. We aimed to quantify circulating extracellular vesicles in pregnant women with type 1 diabetes and to examine associations between extracellular vesicle levels, continuous glucose measures, and pregnancy outcomes.

**Methods:**

We used plasma samples from the Continuous Glucose Monitoring in Women with Type 1 Diabetes in Pregnancy Trial study and quantified circulating extracellular vesicles by flow cytometry (*n* = 163). Relationships with clinical variables were assessed by repeated measures correlation. Logistic regression was used to assess associations between elevated extracellular vesicle levels and pregnancy outcomes.

**Results:**

Platelet extracellular vesicle levels were inversely associated with glucose time above range and glycaemic variability measures (*P* < 0.05). A weak positive association was observed between endothelial extracellular vesicles and mean amplitude of glycemic excursion (*P* < 0.05). In a univariate logistic regression model, high baseline endothelial extracellular vesicles was associated with increased risk of neonatal intensive care unit (NICU) admission (OR: 2.06, 1.03–4.10), and respiratory distress requiring ventilation (OR: 4.98, 1.04–23.92). After adjusting for HbA1c and blood pressure the relationship for NICU admission persisted and an association with hyperbilirubinemia was seen (OR: 2.56, 1.10–5.94). Elevated platelet extracellular vesicles were associated with an increased risk of NICU admission (OR: 2.18, 1.04–4.57), and hyperbilirubinemia (OR: 2.61, 1.11–6.12) after adjusting for HbA1c and blood pressure.

**Conclusions:**

High levels of extracellular vesicles in early pregnancy were associated with adverse neonatal outcomes. Assessment of extracellular vesicles may represent a novel approach to personalized care in type 1 diabetes pregnancy.

**Supplementary Information:**

The online version contains supplementary material available at 10.1186/s40364-021-00322-8.

## Background

Maternal vascular health is a critical determinant of pregnancy outcomes and endothelial injury/dysfunction may play a causal role in acute and longer-term maternal fetal health outcomes [1, 2]. Biomarkers of vascular injury, for example, soluble intercellular adhesion molecule-1 and soluble vascular cell adhesion molecule-1 elevations, are associated with preterm delivery [3]. Similarly, an increase in serum levels of the anti-angiogenic sFlt-1 is associated with a higher rates of early and late preterm births, low birth weight and preeclampsia [4]. Impairment in endothelial function (assessed by flow-mediated dilatation) is observed in women with preeclampsia as compared to women without complications which highlights the important role of the endothelium in disease pathogenesis [5, 6]. Given the importance of the maternal vasculature to both maternal and fetal health, the assessment of maternal vascular health may aid in identification of individuals at greatest risk of complications and allow for intervention with strategies to improve vascular health [7]. For example, the use of real time continuous glucose monitoring has been shown to improve maternal and neonatal outcomes, however its effect on maternal vascular health is not known.

One emerging approach for the assessment of vascular health is enumeration of circulating extracellular vesicles (EVs). EVs are membrane-enclosed vesicles lacking replicative capacity that are released into the extracellular environment and involved in cell signaling [8]. A subclass of large EVs (L-EVs) historically termed microparticles/microvesicles are ~ 100-1000nm in size and shed from the surface of cellular membranes under stress conditions [8, 9]. EVs are comprised of miRNA, mRNA and membrane and cytosolic protein but generally lack nuclear material [8]. EVs exist in numerous biological fluids such as urine, blood plasma, ascites and sputum [8, 9]. Circulating EVs have been extensively studied and primarily originate from platelets, endothelial cells, and leukocytes [10, 11]. Notably, levels of circulating endothelial EVs demonstrate strong inverse correlations with arterial flow-mediated dilatation, and positive correlations with systolic blood pressure and pulse wave velocity [12, 13]. Elevated circulating endothelial EVs, as assessed by flow cytometry, have been shown to predict risk of adverse cardiovascular events independent of traditional risk factors [14, 15].

In diabetes, studies have consistently observed elevated levels of circulating EVs (particularly endothelial EVs) among individuals with type 1 and type 2 diabetes [16, 17]. Sabatier and colleagues revealed that individuals with type 1 diabetes have higher numbers of circulating endothelial, platelet, and total annexin V–positive EVs and increased EV procoagulant activity in comparison with age-matched control subjects [17]. Similarly, Li, S. et al. observed that platelet and monocyte EVs are elevated in individuals with type 2 diabetes [18]. Improved glycemia is associated with reduced levels of circulating EVs in individuals with type 2 diabetes after bariatric surgery [19]. Thus, assessment of EV levels may reflect dynamic changes to the vasculature in diabetes.

To date, the predictive value of circulating EVs in pregnancy is unclear. The aim of our study was to quantify circulating EVs in pregnant women with type 1 diabetes and to examine the associations between EV levels with glycaemia, blood pressure and pregnancy outcomes. We hypothesized that levels of endothelial EVs would be positively correlated with glucose levels and that high levels would be associated with adverse pregnancy outcomes. A secondary goal of this work was to determine whether the use of real time continuous glucose monitoring alters levels of circulating EVs.

## Methods

### Study participants and ethics

We examined plasma samples from the Continuous Glucose Monitoring in Pregnant Women with Type 1 Diabetes (CONCEPTT) trial bioreposity [20]. Details of the clinical study protocol have been previously published [20, 21]. In brief, women with type 1 diabetes who were pregnant or planning pregnancy were randomized to receive real-time continuous glucose monitoring (CGM) or standard care. Those receiving standard care wore a masked CGM at 4–12, 24 and 34 weeks gestation. We conducted a secondary analysis on biospecimens from all 163 pregnant participants with at least one plasma sample available for analysis. Samples from collections at 4–12 weeks (baseline), 24, and 34 weeks gestation were studied. A summary of baseline characteristics and pregnancy outcomes is shown in Table 1. The study was approved by the Mount Sinai Research Ethics Board (ID#: 17-0066-E) and the Ottawa Hospital Research Ethics Board (ID#: 20170658-01 H). Samples were analyzed in a blinded fashion.

**Table 1:** Baseline characteristics and pregnancy outcomes

**Characteristic**^a^	
Age- years	31.4 ± 4.8
BMI- kg/m²	25.7 ± 0.3
European/Mediterranean origin	141 (86.5 %)
Duration of diabetes- years	16.7 ± 7.9
Presence of diabetes complications	50 (30.7 %)
Nulliparity	66 (40.4 %)
Systolic blood pressure- mmHg	115.3 ± 16.1
Diastolic blood pressure- mmHg	69.8 ± 9.9
History of preeclampsia	10 (6 %)
**Maternal outcome**	
Hypertension (Worsening Chronic, Gestational or Pre-eclampsia)	37 (22.7 %)
Preeclampsia	20 (12.3 %)
Gestational Hypertension	15 (9.2 %)
New onset proteinuria	25 (15.3 %)
Episodes of severe hypoglycemia	4 (2.5 %)
Impaired liver function	15 (9.2 %)
Vaginal labour	54 (33.1 %)
Caesarean labour	104 (63.8 %)
Maternal complications	22 (13.5 %)
Maternal length of hospital stay- days	4.9 ± 3.5
**Neonatal Outcome**	
Early preterm (< 34 weeks)	10 (6.3 %)
Late preterm (34–37 weeks)	53 (32.5 %)
Birthweight- g	3553 ± 737
Gestational age at delivery- weeks	37.0 ± 2.0
Neonatal length of hospital stay (days)	5.5 ± 5.8
Neonatal intensive care unit admission	52 (31.9 %)
Neonatal hypoglycaemia	35 (21.5 %)
Composite fetal outcome	70 (43 %)
Respiratory distress	11 (6.8 %)
Antenatal corticosteroids (y/n)	41 (25 %)

^a^values are mean ± SD or n (%) as appropriate

### Continuous glucose monitoring metrics

Summary metrics for CGM measures were obtained for baseline, 24, and 34 weeks gestation. Measures included the mean CGM glucose level, the percentage of time spent within the pregnancy glucose target range (TIR, 63–140 mg/dL), and time spent above (TAR, > 140 mg/dL) and below (TBR, 63 mg/dL) the target range, mean amplitude of glycemia excursion (MAGE), and measures of glycemic variability (SD and coefficient of variation [CV]).

### Isolation and immunolabeling of EVs from archived plasma

We isolated circulating L-EVs (often referred to as microparticles) from archived platelet-free plasma as described previously [22]. Plasma samples were thawed quickly in a water bath at 37 °C. One hundred seventy-five microliter of samples were centrifuged at 12,000 × g for 2 min at 4 °C. One hundred fifty microliter of the supernatant was transferred to fresh tubes and centrifuged at 20,000 x g for 20 min. The supernatant was discarded and L-EV pellets were re-suspended in 150 µl of Annexin V binding buffer (10mM HEPES 140 mM NaCl, 2.5 mM CaCl2, pH 7.4). In fresh tubes, 30 µl of the samples were diluted in Annexin V binding buffer to a final volume of 200 µl for platelet labelling. The platelet samples labelling was performed using anti-CD41-APC (1:100) and Annexin V- FITC (1:50) as labels. Endothelial/leukocytes labelling was conducted by diluting 120 µl of resuspended samples in Annexin V binding buffer to a final volume of 200 µl. Anti-CD144-PE (1:100), anti-CD45-BV421 (1:25) and Annexin V-FITC (1:50) were used to label endothelial/leukocytes samples. After labeling, samples were re-centrifuged at 20,000 g x 20 min after a two hours incubation in the dark. The supernatant was removed, and pellet was preserved and re-suspended in Annexin Binding Buffer (300 µL). The samples were transferred to flow cytometry tubes for analysis. All isotype controls, antibodies and Annexin V were purchased from BioLegend (San Diego, USA).

### Quantitation of circulating EVs by nanoscale flow cytometry

L-EVs were quantified at the University of Ottawa Flow Cytometry and Virometry Facility using a Beckman Coulter CytoFLEX S with CyExpert Version 2.3.0.84. ApogeeMix beads (Apogee Flow Systems, Hertfordshire, UK) were used to establish a size gate of ~ 100–1000 nm. L-EVs were identified as ~ 100–1000 nm particles in size staining positive for the membrane marker Annexin V compared to negative controls. Results are expressed as the number of annexinV+ (Total), annexin V + and CD41+ (platelet), annexin V + and CD45+ (leukocyte) or annexin V + and CD144+ (endothelial) EVs/ mL plasma. Samples labelled with isotype controls, antibodies alone in buffer, and unlabelled samples were analyzed as controls. FlowJo ver 7.6.5 was used for analysis.

### Statistical analysis

Direct comparisons between EV levels were conducted by t-test or ANOVA (as appropriate) using GraphPad Prism version 9 (GraphPad Software). 

Univariate and multivariate logistic regression models were tested using SPSS version 26 (IBM Corp, Armonk, NY). Logistic regression models with baseline EV levels as a dichotomous variable (above and below median value) were used to evaluate relationships with maternal and neonatal outcomes. Multivariate logistic regression models were designed *a priori* to adjust for known risk factors that have been shown to correlate with circulating EV levels in the general population (factors included were: systolic and diastolic blood pressure and HbA1c%.) Relationships between EV levels and continuous variables throughout the study were assessed by repeated measures correlation in R 3.5.1 using the repeated measures correlation (rmcorr) package version 0.4.0 [23].

All graphs were constructed using GraphPad Prism. A *P* value < 0.05 was considered significant.

## Results

### EV levels throughout pregnancy

We first examined levels of circulating platelet, endothelial, leukocyte, and total EVs to determine if levels change throughout type 1 diabetes pregnancy. We did not observe significant differences in levels of endothelial (*P* = 0.43), leukocyte (*P* = 0.65), platelet (*P* = 0.82), or total EVs (*P* = 0.58) between trimesters (Supplemental Fig. 1).

### Association between circulating EVs and glycemic variables

We next analyzed relationships of EVs with HbA1c and continuous glucose measures throughout pregnancy by repeated measures correlation (Table 2). We did not observe significant associations between EV populations and mean glucose (24 h, obtained from CGM data) or HbA1c. A weak positive correlation between endothelial EVs and MAGE was observed but this was not accompanied by associations with other glycemic variability markers including CV and SD. Interestingly, we observed an inverse relationship between platelet EV levels and glucose SD, MAGE and TAR as well as a positive correlation with TIR suggesting an inverse relationship between platelet EVs and glucose levels. No relationships between leukocyte EVs and HbA1c or continuous glucose measures were observed.

**Table 2:** Association between circulating endothelial EVs and clinical variables by repeated measures correlation

EV population	Clinical Variable	r	95% CI	***p***
**Endothelial**	HbA1c	0.040	-0.072, 0.164	0.44
	Mean CGM Glucose	0.091	-0.006, 0.186	0.06
	% TIR (63-140mg/dl)	-0.005	-0.129, 0.118	0.94
	% TAR( >140mg/dl)	0.064	-0.060, 0.187	0.31
	% TBR (<63mg/dl)	-0.116	-0.236, 0.008	0.07
	Glucose CV	-0.012	-0.135, 0.112	0.85
	Glucose SD	0.054	-0.070, 0.177	0.39
	**MAGE**	**0.129**	**0.005, 0.248**	**0.04**
	SBP	0.082	-0.035, 0.196	0.17
	DBP	0.075	-0.043, 0.189	0.21
**Platelet**	HbA1c	0.009	-0.107, 0.126	0.88
	Mean CGM Glucose	-0.111	-0.231, 0.013	0.08
	**% TIR (63-140mg/dl)**	**0.159**	**0.035, 0.276**	**0.01**
	**% TAR( >140mg/dl)**	**-0.138**	**-0.257, -0.014**	**0.03**
	% TBR (<63mg/dl)	-0.055	-0.178, 0.068	0.38
	Glucose CV	-0.109	-0.229, 0.015	0.08
	**Glucose SD**	**-0.147**	**-0.267, -0.025**	**0.02**
	**MAGE**	**-0.152**	**-0.271, -0.029**	**0.01**
	SBP	0.051	-0.066, 0.167	0.39
	DBP	0.099	-0.017, 0.210	0.09
**Leukocyte**	HbA1c	0.001	-0.116, 0.116	0.99
	Mean CGM Glucose	0.003	-0.120, 0.127	0.95
	% TIR (63-140mg/dl)	0.007	-0.116, 0.131	0.91
	% TAR( >140mg/dl)	0.003	-0.121, 0.127	0.96
	% TBR (<63mg/dl)	-0.030	-0.154, 0.093	0.62
	Glucose CV	0.008	-0.115, 0.132	0.89
	Glucose SD	0.013	-0.110, 0.137	0.83
	MAGE	0.001	-0.124, 0.123	0.99
	**SBP**	**0.179**	**0.064, 0.289**	**0.002**
	**DBP**	**0.200**	**0.093, 0.315**	**0.001**

Significant relationships are bolded

*CGM* continuous glucose monitoring, *TIR* time in range, *TAR* time above range, *TBR* time below range, *CV* coefficient of variation, *SD* standard deviation, *MAGE* mean amplitude of glycemic excursion, *SBP* systolic blood pressure, *DBP* diastolic blood pressure

We also examined the association between EV levels and blood pressure by repeated measures correlation. There was no correlation between endothelial or platelet EVs with either systolic or diastolic blood pressure. However, we did observe a positive correlation between leukocyte EVs and both systolic and diastolic blood pressure (Table 2).

### Effect of CGM on circulating EV

**Fig. 1:**
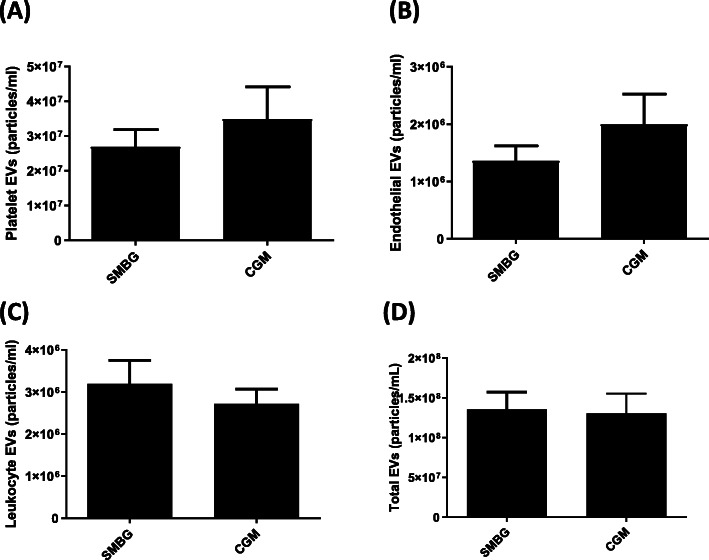
The effect of RT-CGM on levels of circulating EVs. Shown are values of circulating levels of EVs at 34 weeks gestation in individuals utilizing real time continuous glucose monitoring (CGM) compared with conventional self-monitoring of blood glucose (SMBG). **A** Platelet-derived EV; **B**. Endothelial-derived EV; **C**. Leukocyte-derived EV and **D**. Total annexin + EVs. The number of participants for CGM and SMBG are *n* = 72 and *n* = 77 respectively. No significant differences were observed for platelet-derived EVs (0.74), endothelial-derived EV (0.88), leukocyte-derived EVs (0.31), or total annexin V + EVs (0.17)

We next assessed whether using CGM compared with self-monitoring of blood glucose (SMBG) had an effect on circulating EVs. Figure 1 shows the effect of use of CGM on platelet, endothelial, leukocyte, and total EV levels at 34 weeks gestation. No differences between CGM and SMBG were observed for circulating endothelial, platelet, leukocyte, or total EVs at 34 weeks gestation.

### EVs and pregnancy outcomes

**Fig. 2:**
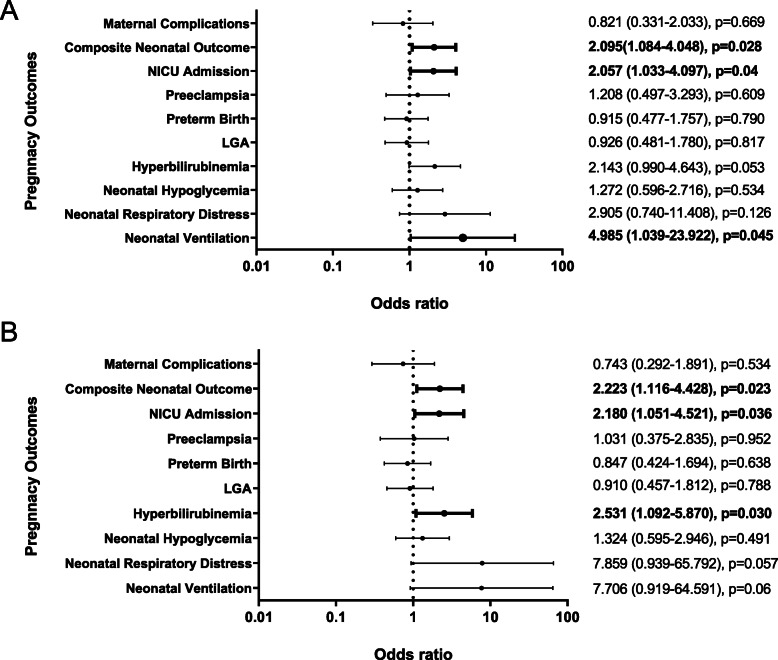
High baseline endothelial EV levels are associated with adverse pregnancy outcomes. **A**: Forest plot depicting the relationship between baseline endothelial EV levels and pregnancy outcomes. **B**: Forest plot depicting the relationship between baseline endothelial EV levels and pregnancy outcomes after adjusting for the HbA1c and systolic and diastolic blood pressure. Maternal complications indicates the presence of oliguria, cerebral of visual symptoms, impairment of liver function, pulmonary edema or cyanosis, epigastric or right upper quadrant pain, or thrombocytopenia. Composite Neonatal Outcome: aggregate measure of pregnancy loss, birth injury, neonatal hypoglycaemia, and respiratory distress; LGA: Large for gestational age (> 90 percentile); Ventililation: respiratory distress requiring positive pressure ventilation

Finally, given that EV levels are reflective of vascular health, we assessed whether baseline levels of EVs were predictive of pregnancy outcomes. In a univariate logistic regression model, we observed that higher baseline levels of endothelial EVs (above median) were associated with increased risk of neonatal intensive care unit (NICU) admission, respiratory distress requiring positive pressure ventilation, and positivity for the composite fetal outcome (an aggregate of pregnancy loss, birth injury, neonatal hypoglycaemia, and respiratory distress) [Fig. 2A]. After adjusting for HbA1c, systolic and diastolic blood pressure, we observed significant positive associations with NICU admission, composite neonatal outcome, and hyperbilirubinemia (Fig. 2B). A weak positive association between baseline endothelial EV levels and maternal length of hospital stay was also observed (*p* = 0.03, *r* = 0.17).

**Fig. 3:**
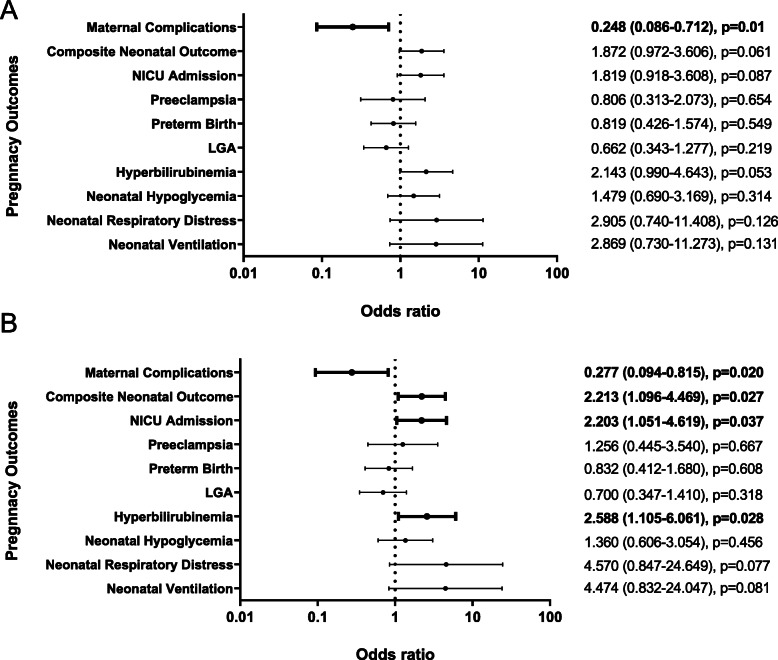
Relationship between platelet EV level and pregnancy outcomes. **A**: Forest plot depicting the relationship between baseline platelet EV levels and pregnancy outcomes. **B**: Forest plot depicting the relationship between baseline platelet EV levels and pregnancy outcomes after adjusting for the baseline HbA1c and systolic and diastolic blood pressure

With respect to platelet EVs, we observed that low levels of platelet EVs were associated with maternal complications in the unadjusted model (Fig. 3A). After adjustment for baseline HbA1c and blood pressure, platelet EVs were inversely associated with maternal complications (Fig. 3B). By contrast positively associations with neonatal intensive care unit admission, hyperbilirubinemia, and composite fetal outcome (Fig. 3B). We did not observe any relationships between leukocyte EVs and pregnancy outcomes (data not shown).

## Discussion

Our major finding is that baseline levels of circulating EVs are higher in women with neonatal complications. After adjustment for known risk factors (HbA1c and blood pressure), higher baseline endothelial EVs were associated with increased NICU admission and hyperbilirubinemia. Similarly, higher baseline platelet EVs were associated with increased risk of NICU and hyperbilirubinemia but decreased maternal complications after adjustment for HbA1c and blood pressure.

It is well established that glucose is a potent stimulus for EV formation. We have shown that high glucose induces L-EV release from cultured endothelial cells [24] and podocytes [25] and that acute hypoglycemia increases urinary podocyte EV levels [26]. Similarly, mesangial cells release more EVs in response to high glucose stimulation [27] and animal and clinical studies consistently show elevations in circulating EVs in diabetes [16–19]. Surprisingly, we did not observe any correlation between circulating EVs and HbA1c or mean glucose. We did observe a weak positive correlation between endothelial EVs and MAGE but other measures of glucose variability (SD, CV) did not show the same relationship. Similarly, we did not observe any associations between endothelial EVs and blood pressure values. Thus, while glucose and blood pressure may influence endothelial EV levels in type 1 diabetes in pregnancy, other factors are also contributing to EV dynamics. Perhaps more surprising is the observation that platelet EVs were inversely correlated with MAGE, SD, and time above range and positively correlated with time in range. These data suggest that platelet EV formation may be reduced with hyperglycaemia in pregnancy and are in contrast to studies outside of pregnancy including a systematic review which reported that platelet EVs are increased in hyperglycaemic conditions [17, 18]. The reason for this discrepancy is unclear although ours is the first study to examine this relationship in type 1 diabetes in pregnancy. Two previous studies, performed 15–20 years ago in normal, hypertensive and growth restricted pregnancies, reported reduced platelet EV levels in preeclampsia despite evidence of platelet activation [28, 29] so it is possible that the molecular mechanisms underlying platelet EV formation are altered in pregnancy. Further study is needed to better understand the relationship between glucose levels and platelet EVs in pregnancies complicated by diabetes.

The use of CGM in individuals with type 1 and type 2 diabetes is well established to improve glycemic control and it has been speculated that the improved glycemia associated with CGM, may reduce microvascular complications [30–32]. Our laboratory and others have reported that endothelial EVs are biomarkers of glucose-induced endothelial injury [17, 24]. Given this, we hypothesized that CGM, which was associated with improved glucose levels in the randomized controlled trial [20], would lead to reduced circulating endothelial EVs. However, we did not observe any differences in circulating EVs between the SMBG control and CGM intervention groups. Given the modest improvement in glucose levels, this secondary analysis was likely not powered to detect between-group differences in EVs. In addition, the short duration of the intervention (started in the late first/early second trimester) may not have been sufficient to impact cellular injury and resultant EV formation.

Our most striking observation is that high levels of endothelial and platelet EVs early in pregnancy were associated with adverse neonatal outcomes. Elevated levels of EVs have long been known to predict adverse cardiovascular outcomes in other populations. Sinning et al. [15] showed that high levels of endothelial EVs were associated with increased cardiovascular morbidity and mortality over a 6 year follow-up. Similarly, Amabile et al. revealed that endothelial EVs are elevated in individuals with pulmonary hypertension and directly linked to hemodynamic severity of this condition [14]. In the present study we found that high levels of endothelial EVs (above median value) were associated with increased NICU admission, neonatal respiratory distress requiring positive pressure ventilation, and hyperbilirubinemia. Similarly, high levels of platelet EVs, after adjustment for HbA1c and blood pressure, were associated with NICU admission, neonatal resuscitation, hyperbilirubinemia and positivity for the composite fetal outcome. Curiously, low levels of platelet EVs also seemed to predict maternal complications in this cohort. Of note is the observation that high levels of EVs were not predictive of preeclampsia in this population in contrast to other studies which found that EVs were increased in preeclampsia [33, 34]. This may be related to the sample size, low number of cases in our study population and differences between normal, hypertensive and diabetes pregnancies.

Endothelial EVs are also engaged in pathological processes as they are able to induce vascular injury [24, 35–37]. Previous studies have shown that EVs induce endothelial inflammation, haemostasis, angiogenesis, and endothelial dysfunction [35]. Thus, in addition to serving as biomarkers of vascular injury they may actually induce vascular dysfunction through direct actions on endothelial cells leading to increased oxidative stress, inflammation, and impaired endothelium-dependent vasorelaxation [24, 36–40]. We and others have previously shown that endothelial EVs exert greater deleterious effects on the endothelium when formed under hyperglycaemic conditions [24, 41]. Bidirectional EV-mediated cross-talk between the maternal vasculature and placenta may also play an important role in disease pathogenesis. In this regard, Kohli et al. have shown that maternal EVs can promote inflammatory responses in trophoblasts [42] and Han et al. recently reported that placental-derived EVs can induce preeclamptic symptoms in mice [43]. Whether this is true in diabetes pregnancy and whether increased EVs played a causal role in the pregnancy-related complications observed in the present study is a subject for future investigation.

One of the strengths of this study is the use of human plasma samples from a multicenter, randomized controlled trial with extensive participant characterization. In addition, while many studies focus on a single EV population, our flow cytometry approach examined EVs from endothelial cells, leukocytes, and platelets. Indeed, we were able to identify predictive value for both endothelial and platelet EVs. In addition, the availability of CGM data allowed for assessment of relationships between EVs and glycaemia to an extent that has not been done previously. Nevertheless, our study also has limitations: First, while this represents a large cohort in the context of prior work, it is still a relatively small sample with limited neonatal complications. Second, relationships between EV levels were assessed at only three time points and this may have limited our ability to identify associations with clinical variables. Finally, our cohort was a secondary analysis of a clinical trial focused exclusively on pregnant women with type 1 diabetes. As such we were unable to compare values to those without diabetes or those with type 2 or gestational diabetes. Given differences in sample collection methods, interlaboratory differences in sample preparation, analysis, and instrument sensitivity numeric comparisons to previous data is challenging, however we note that the levels of endothelial and platelet EVs seen in our study are slightly higher than previous reports in non-pregnant healthy donors, individuals with type 1 or type 2 diabetes or in non-diabetic pregnant individuals [16, 17, 44]. However direct comparison may not be appropriate as these differences could be due to greater assay sensitivity.

## Conclusions

In summary, our results suggest that elevated endothelial or platelet EVs are associated with perinatal complications in pregnant women with type 1 diabetes. Accordingly, assessment of EV levels early in pregnancy may aid in identifying women at high risk of neonatal complications.

## Supplementary Information

**Additional file 1: Supplemental Figure 1. **The effect of RT-CGM on levels of circulating EVs. Shown are values of circulating levels of EVs at 34 weeks gestation in individuals utilizing real time continuous glucose monitoring (CGM) compared with conventional self-monitoring of blood glucose (SMBG). (A) Platelet-derived EV; (B). Endothelial-derived EV; (C). Leukocyte-derived EV and (D). Total annexin V levels. The number of participants for CGM and SMBG are *n *= 72 and *n *= 77 respectively. No significant differences were observed for platelet-derived EVs (0.74), endothelial-derived EV (0.88), leukocyte-derived EVs (0.31), or total annexin V+ EVs (0.17).

**Additional file 2. List of members of the CONCEPTT Collaborative Group**

## Data Availability

The datasets used and/or analysed during the current study are available from the corresponding authors on reasonable request.

## Abbreviations

EVs: Extracellular vesicles
L-EVs: Largeextracellular vesicles
CONCEPTT: Continuous Glucose Monitoring in Pregnant Women with Type 1 Diabetes
CGM: Continuous glucose monitoring
TIR: Time in range
TAR: Time spent above range
TBR: Time spent below range
MAGE: Mean amplitude of glycemia excursion
SD: Standard deviation
CV: Coefficient of variation
SMBG: Self-monitoring of blood glucose
NICU: Neonatal intensive care unit admission
T1D: Type 1 diabetes
T2D: Type 1 diabetes
