# Supplementary material for: Circulating extracellular vesicles during pregnancy in women with type 1 diabetes: a secondary analysis of the CONCEPTT trial
Source: Biomark Res. 2021 Sep 6;9:67. doi: 10.1186/s40364-021-00322-8 (PMC8419913; doi:10.1186/s40364-021-00322-8)
Supplement: Supplementary file 1 — Additional file 1: Supplemental Figure 1. The effect of RT-CGM on levels of circulating EVs. Shown are values of circulating levels of EVs at 34 weeks gestation in individuals utilizing real time continuous glucose monitoring (CGM) compared with conventional self-monitoring of blood glucose (SMBG). (A) Platelet-derived EV; (B). Endothelial-derived EV; (C). Leukocyte-derived EV and (D). Total annexin V levels. The number of participants for CGM and SMBG are n = 72 and n = 77 respectively. No significant differences were observed for platelet-derived EVs (0.74), endothelial-derived EV (0.88), leukocyte-derived EVs (0.31), or total annexin V+ EVs (0.17). [file 40364_2021_322_MOESM1_ESM.pptx]

## Slide 1
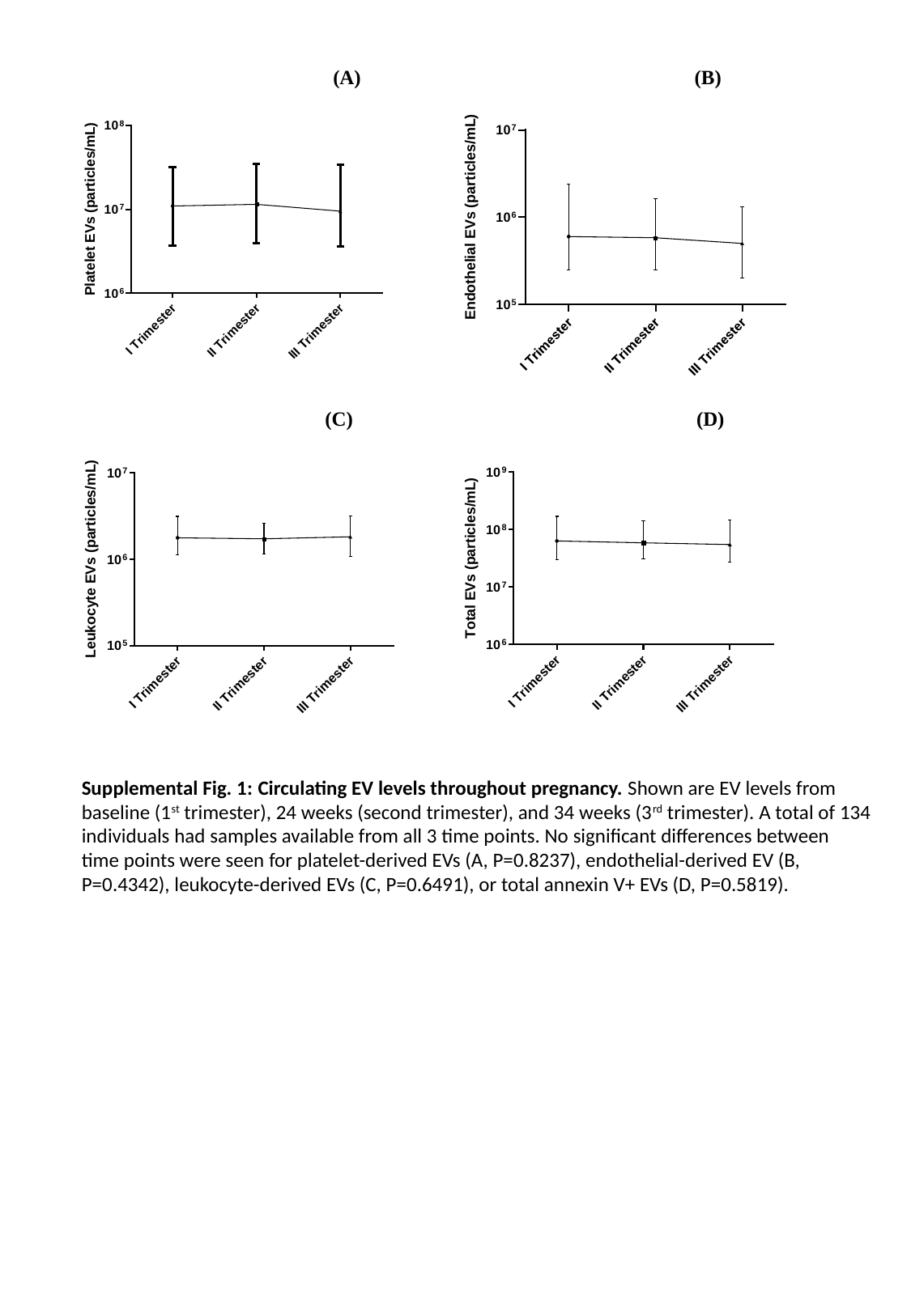

(A) (B)
(C) (D)
Supplemental Fig. 1: Circulating EV levels throughout pregnancy. Shown are EV levels from baseline (1st trimester), 24 weeks (second trimester), and 34 weeks (3rd trimester). A total of 134 individuals had samples available from all 3 time points. No significant differences between time points were seen for platelet-derived EVs (A, P=0.8237), endothelial-derived EV (B, P=0.4342), leukocyte-derived EVs (C, P=0.6491), or total annexin V+ EVs (D, P=0.5819).
