# Supplementary material for: Circulating extracellular vesicles during pregnancy in women with type 1 diabetes: a secondary analysis of the CONCEPTT trial
Source: Biomark Res. 2021 Sep 6;9:67. doi: 10.1186/s40364-021-00322-8 (PMC8419913; doi:10.1186/s40364-021-00322-8)
Supplement: Supplementary file 2 — Additional file 2. List of members of the CONCEPTT Collaborative Group [file 40364_2021_322_MOESM2_ESM.docx]

CONCEPTT Collaborative Group (listed according to recruitment numbers): Cambridge University Hospitals NHS Foundation Trust, Cambridge, UK: Helen Murphy, Jeannie Grisoni, Carolyn Byrne, Sandra Neoh, Katy Davenport, (43); Alberta Health Services, University of Calgary, Calgary, Canada: Lois Donovan, Claire Gougeon, Carolyn Oldford, Catherine Young (39); King’s College Hospital, London, UK: Stephanie Amiel, Katharine Hunt, Louisa Green, Helen Rogers, Benedetta Rossi (29); Mount Sinai Hospital, Toronto, Canada: Denice Feig, Barbara Cleave, Michelle Strom (22); Hospital de la Santa Creu i Sant Pau, Barcelona, Spain and CIBER‐BBN, Zaragoza, Spain: Rosa Corcoy, Alberto de Leiva, Juan María Adelantado, Ana Isabel Chico, Diana Tundidor (22); The Ottawa Hospital General Campus, Ottawa, Canada: Erin Keely, Janine Malcolm, Kathy Henry (15); Ipswich Hospital NHS Trust, Ipswich, UK: Damian Morris, Gerry Rayman, Duncan Fowler, Susan Mitchell, Josephine Rosier (13); Norfolk and Norwich University Hospital, Norwich, UK: Rosemary Temple, Jeremy Turner, Gioia Canciani, Niranjala Hewapathirana, Leanne Piper (13); St. Joseph's Health Centre, London, Canada: Ruth McManus, Anne Kudirka, Margaret Watson (13); Niguarda ca’ Granda Hospital, Milano, Italy: Matteo Bonomo, Basilio Pintaudi, Federico Bertuzzi, Giuseppina Daniela Corica, Elena Mion (12); Sunnybrook Health Sciences Centre, Toronto, Canada: Julia Lowe, Ilana Halperin, Anna Rogowsky, Sapida Adib (11); Glasgow Royal Infirmary, Glasgow, UK: Robert Lindsay, David Carty, Isobel Crawford, Fiona Mackenzie, Therese McSorley (10); McMaster University, Hamilton, Canada: John Booth, Natalia McInnes, Ada Smith, Irene Stanton, Tracy Tazzeo (8); Centre hospitalier universitaire de Québec, Quebec City, Canada: John Weisnagel (6); Queen’s Medical Centre, Nottingham, UK: Peter Mansell, Nia Jones, Gayna Babington, Dawn Spick (6); Royal Victoria Infirmary, Newcastle Upon Tyne, Newcastle, UK: Malcolm MacDougall, Sharon Chilton, Terri Cutts, Michelle Perkins (6); Leeds Teaching Hospitals NHS Trust, Leeds, UK: Eleanor Scott, Del Endersby (6); Royal Infirmary of Edinburgh, Edinburgh, UK: Anna Dover, Frances Dougherty, Susan Johnston (6); Sheffield Teaching Hospitals NHS Foundation Trust, Sheffield, UK: Simon Heller, Peter Novodorsky, Sue Hudson, Chloe Nisbet (6); Izaak Walton Killam Health Sciences Centre (IWK), Halifax, Canada: Thomas Ransom, Jill Coolen, Darlene Baxendale (5); University Hospital Southampton NHS Foundation Trust, Southampton, UK: Richard Holt, Jane Forbes, Nicki Martin, Fiona Walbridge (6); Galway University Hospitals, Galway, Ireland: Fidelma Dunne, Sharon Conway, Aoife Egan, Collette Kirwin (4); Central Manchester University Hospitals NHS Foundation Trust, Manchester, UK: Michael Maresh, Gretta Kearney, Juliet Morris, Susan Quinn (4); South Tees Hospitals, NHS Foundation Trust, Middlesbrough, UK: Rudy Bilous, Rasha Mukhtar (4); Centre de Recherche du Centre Hospitalier de Université de Montréal (CR‐CHUM), Montreal, Canada: Ariane Godbout, Sylvie Daigle (3); The Dudley Group NHS FT, Russells Hall Hospital, Dudley, UK: Alexandra Lubina Solomon, Margaret Jackson, Emma Paul, Julie Taylor (3); Kingston General Hospital, Queen’s University, Kingston, Canada: Robyn Houlden, Adriana Breen (3); Guys and St Thomas’ NHS Foundation Trust, London, UK: Anita Banerjee, Anna Brackenridge, Annette Briley, Anna Reid, Claire Singh (2); Royal University Hospital, Saskatoon, Canada: Jill Newstead‐Angel, Janet Baxter (2); Grampian Diabetes Centre, Aberdeen, UK: Sam Philip, Martyna Chlost, Lynne Murray (2); William Sansum Diabetes Center, Santa Barbara, USA: Kristin Castorino, Lois Jovanovic, Donna Frase (2). The Centre for Clinical Trial Support (CCTS) at the Sunnybrook Research Institute, Toronto, Canada: Sonya Mergler, Kathryn Mangoff, Johanna Sanchez, and Gail Klein. The Jaeb Center for Health Research, Tampa, USA: Katrina Ruedy and Craig Kollman. Juvenile Diabetes Research Foundation (non‐clinical collaborators): Olivia Lou and Marlon Pragnell.
